# SOX2 and OCT4 mediate radiation and drug resistance in pancreatic tumor organoids

**DOI:** 10.1038/s41420-024-01871-1

**Published:** 2024-03-01

**Authors:** Sanjit Roy, Tijana Dukic, Zachery Keepers, Binny Bhandary, Narottam Lamichhane, Jason Molitoris, Young H. Ko, Aditi Banerjee, Hem D. Shukla

**Affiliations:** 1 Division of Translational Radiation Sciences, Department of Radiation Oncology, University of Maryland School of Medicine, Baltimore, MD USA; 2 Department of Pediatrics, University of Maryland School of Medicine, Baltimore, MD USA

**Keywords:** Oncogenesis, Cancer stem cells

## Abstract

Pancreatic cancer has a five-year survival rate of only 10%, mostly due to late diagnosis and limited treatment options. In patients with unresectable disease, either FOLFIRINOX, a combination of 5-fluorouracil (5-FU), oxaliplatin and irinotecan, or gemcitabine plus nab-paclitaxel combined with radiation are frontline standard regimens. However, chemo-radiation therapy has shown limited success because patients develop resistance to chemotherapy and/or radiation. In this study, we evaluated the role of pancreatic cancer stem cells (CSC) using OCT4 and SOX2, CSC markers in mouse pancreatic tumor organoids. We treated pancreatic tumor organoids with 4 or 8 Gy of radiation, 10 μM of 5-FU (5-Fluorouracil), and 100 μM 3-Bromopyruvate (3BP), a promising anti-cancer drug, as a single treatment modalities, and in combination with RT. Our results showed significant upregulation of, OCT4, and SOX2 expression in pancreatic tumor organoids treated with 4 and 8 Gy of radiation, and downregulation following 5-FU treatment. The expression of CSC markers with increasing treatment dose exhibited elevated upregulation levels to radiation and downregulation to 5-FU chemotherapy drug. Conversely, when tumor organoids were treated with a combination of 5-FU and radiation, there was a significant inhibition in SOX2 and OCT4 expression, indicating CSC self-renewal inhibition. Noticeably, we also observed that human pancreatic tumor tissues exhibited heterogeneous and aberrant OCT4 and SOX2 expression as compared to normal pancreas, indicating their potential role in pancreatic cancer growth and therapy resistance. In addition, the combination of 5-FU and radiation treatment exhibited significant inhibition of the β-catenin pathway in pancreatic tumor organoids, resulting in sensitization to treatment and organoid death. In conclusion, our study emphasizes the crucial role of CSCs in therapeutic resistance in PC treatment. We recommend using tumor organoids as a model system to explore the impact of CSCs in PC and identify new therapeutic targets.

## Introduction

Pancreatic cancer (PC) is the fourth deadliest cancer in the US with an estimated 64,050 new cases and 50,550 deaths in 2023 [1]. Owing to the lack of sensitive biomarkers for early diagnosis, the disease is normally diagnosed in the advanced stages, and less than 20% of diagnosed patients have the option to undergo surgery [2]. Despite modest improvements in systemic therapies for pancreatic cancer and the potential value of stereotactic body radiotherapy or chemoradiation, treatment resistance is common and prognosis for patients remains poor. Therefore, it is necessary to further investigate the molecular mechanisms involved in treatment failure [3].

Pancreatic cancer presents many challenges that hinder treatment. One of the major obstacles is dense stroma which creates a physical barrier. The stroma is filled with fibroblasts and inflammatory cells that result in an immunosuppressive tumor microenvironment (TME), making it difficult for the immune system to recognize and eliminate cancer cells [4, 5]. Recent reports have shown that treatment failure in most malignant tumors could be due to cancer stem cells (CSCs) which may mediate chemo and radiation resistance leading to relapse and metastases [6]. CSCs have gained considerable interest as a potential target for cancer therapy due to their association with treatment resistance [7]. Hypoxic areas of tumors are predominantly inhabited by CSCs, which possess self-renewing capabilities [6, 8]. The CSC phenotypes are regulated by 20 different transcription factors, including OCT4 and SOX2 [9]. They have unique characteristics that allow them to adapt to changes in the tumor microenvironment, such as hypoxia, starvation, and anticancer treatments, leading to survival and resistance to therapies [10, 11]. The signaling pathways implicated in CSCs which are essential for self-renewal include Epithelial-Mesenchymal Transition (EMT) and hypoxia. Furthermore, pancreatic CSCs co-inhabit with other cellular components in the hypoxic niche in the tumor microenvironment and therefore, unraveling the connection between CSCs and the TME is crucial to identify molecular pathways involved in resistance [11].

Recent studies show that tumor organoids accurately reflect the in-vivo state and can serve as a model to evaluate responses to different anti-cancer drugs and RT treatments. Patient-derived tumor organoid models can faithfully replicate the original tumors’ genetic and phenotypic characteristics, and the in vivo tumor microenvironments [11]. Importantly, most 3D organoid reports are focused on response to chemotherapy and other targeted agents [12]. However, few studies have used organoid models to investigate the role of CSCs in therapy resistance and tumor recurrence [13]. The current study aims to define the role played by CSCs in the resistance of pancreatic tumors to radiation and chemotherapy. We demonstrate here the increased effectiveness of the combination of chemo and radiation therapy in suppressing CSCs. Furthermore, we highlight the crucial role of the β-catenin pathway, which may clinically contribute to treatment resistance in PDAC.

## Results

### Analysis of drug and radiation treatment response of pancreatic tumor organoids by Optical Metabolic Imaging

**Fig. 1:**
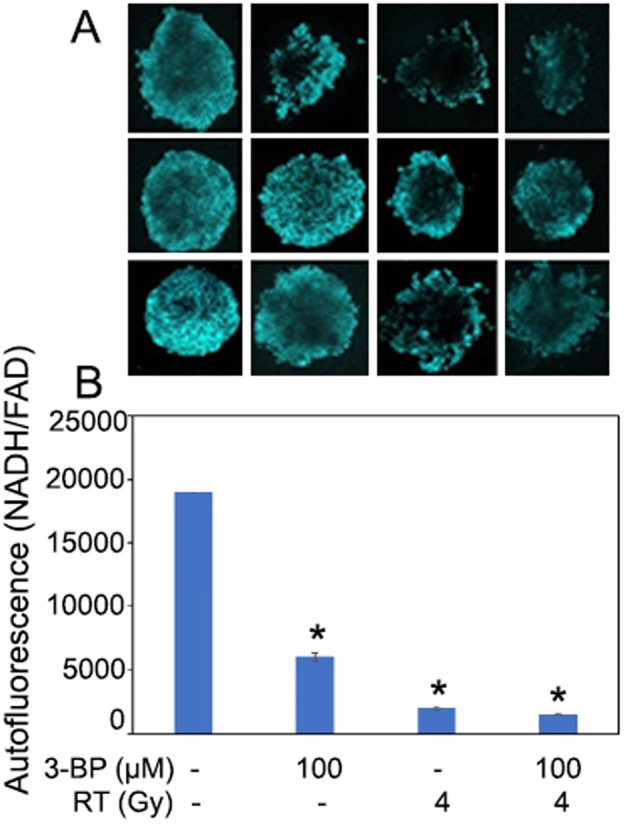
Measurement of radiation and drug treatment response of pancreatic tumor organoids by optical metabolic imaging. **A** Optical metabolic imaging of pancreatic tumor organoids treated with 100 μM of 3-BromoPyruvate, 4 Gy radiation, and combination of both. **B** Quantification of metabolic images as treatment response following the treatment with 3-BP and radiation by ImageJ (*P* < 0.05).

The tumor organoids response to different doses of radiation and 3-BP was examined as the change in autofluorescence of tumor organoids due to metabolic shift. Based on our initial findings, we observed that when tumor organoids were exposed treated with 100 µM of 3-BP for 12 h and 4 Gy of radiation, there was a significant reduction in autofluorescence, and tumor organoid growth compared to the untreated control (Fig. 1A, B). The treatment response was quantified by ImageJ analysis and the data showed that combination treatment inhibited approximately 90% of metabolism in tumor organoids and resulted in tumor organoid death as compared to untreated control (*p* < 0.05). Thus, optical metabolic imaging showed significant inhibition in the growth of tumor organoids treated with combined modalities compared to a single modality treatment. Therefore, the results demonstrated combination treatment was more effective than individual treatment.

### Upregulation of OCT4 and SOX2 expression in tumor organoids treated with 4 and 8 Gy of radiation

**Fig. 2:**
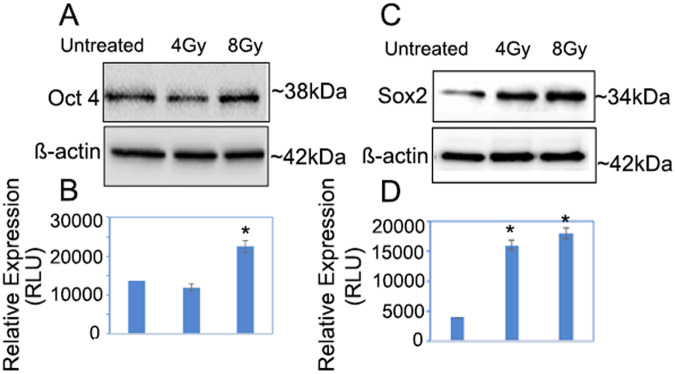
Upregulation of OCT4 and SOX2 in PTOs treated with 4 Gy and 8 Gy radiation. Organoids lysates were analyzed by Western blot for OCT4 (**A**), SOX2 (**C**), and β-actin expression. **B**, **D** Quantification estimations of OCT4 and SOX2 levels determined by densitometry measurements of western blots from three independent experiments after normalization with β-actin (*p* < 0.05).

To further investigate the role of CSCs in chemo-RT resistance, the tumor organoids were passaged and once the size of the organoids reached 100 μm, they were subjected to 4 and 8 Gy of radiation treatment. After the treatment, the organoids were allowed to grow for another 12 h before processing them for immunofluorescence and immunoblot analysis. The results showed upregulation of OCT4, in tumor organoid treated with 8 Gy of radiation (Fig. 2A, B, lanes 3; *P < 0.05). Furthermore, there was also approximately 5-fold upregulation of SOX2 (Fig. 2C, D, lanes 2 and 3; *P* < 0.05), in tumor organoids treated with increasing doses of radiation as compared to control.

### Alteration in OCT4 and SOX2 expression in pancreatic tumor organoids treated with 5-FU chemotherapy drug

**Fig. 3:**
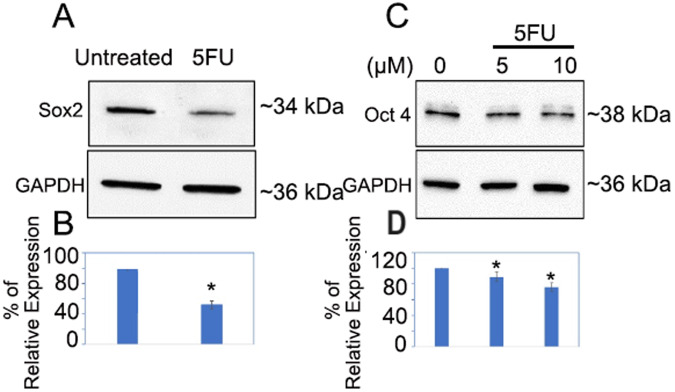
Suppression of OCT4 and SOX2 in PTOs treated with 5-FU chemotherapy drug. **A**, **B** Down regulation of OCT4 & SOX2 in PTOs treated with 5 μM of 5-FU. **C**, **D** Down regulation of OCT4 in PTOs treated with 5 and 10 μM of 5-FU. Quantification of SOX2 and OCT4 was performed by ImageJ software (*p = <0.05).

In contrast, the data revealed that the expression of SOX2 was reduced in PTOs that were treated with 5 µM of 5-FU (Fig. 3A, B). Additionally, we also observed downregulation of OCT4 in PTOs that were treated with 5 and 10 µM of 5-FU. The data demonstrated that there was about a 50% decrease in SOX2 expression in tumor organoids that were treated with 5 µM of 5-FU. Additionally, we found approximately 15 and 20% inhibition of OCT4 in pancreatic tumor organoids that were treated with 5 and 10 μM of 5-FU, respectively, when compared to the untreated control (Fig. 3C, D; *p* < 0.05).

**Fig. 4:**
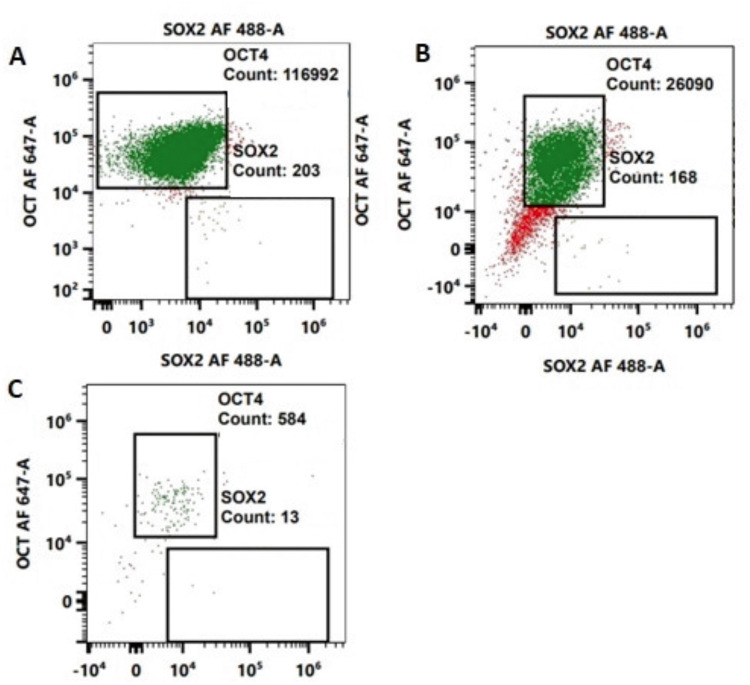
Flow Cytometry analysis of OCT4 and SOX2 in tumor organoids treated with 10 and 50 µM of 5-FU. **A** Untreated tumor organoids. **B** Tumor organoids treated with 10 µM of 5-FU. **C** Tumor organoids treated with 50 µM of 5-FU.

To further validate our data, we also performed Flow Cytometry analysis in pancreatic tumor organoids treated with 5-FU. Our results demonstrated that treatment with 10 μM of 5-FU inhibited the OCT4 expression by 77.69%, while treatment with 50 μM of 5-FU inhibited approx. 99%. Similarly, inhibition of SOX2 marker by 17.24 and 93% following the treatment as compared to untreated control (Fig. 4A–C).

### Combination of chemotherapy and radiation inhibits OCT4 and SOX2 in pancreatic tumor organoids

**Fig. 5:**
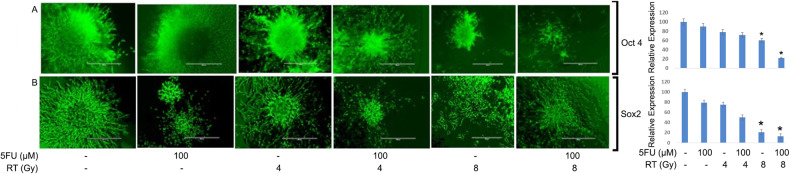
Immunofluorescence staining and quantification of OCT4 and SOX2 in PTOs treated with 5-FU and RT (*p = <0.05). **A** Inhibition of OCT4 in tumor organoids treated with 100 μM of 5-FU, 4 and 8 Gy of RT, and combination of both. **B** Inhibition of SOX2 in tumor organoids treated with 100 μM of 5-FU, 4 and 8 Gy radiation and combination of both.

To investigate the synergistic effects of combination treatments in inhibiting CSCs, we treated pancreatic tumor organoids with individual and combined doses of 5-FU, and radiation. The tumor organoids were first treated with 100 μM of 5-FU and then in combination with 4 Gy and 8 Gy of radiation. After treatment, we performed immunofluorescence staining of OCT4 and SOX2 in tumor organoids to study their expression. The results demonstrated combination of 100 μM of 5-FU and 4 Gy of radiation significantly suppressed OCT4 and SOX2 expression and inhibited tumor organoid growth as compared to untreated control (Fig. 5A, B; *p* < 0.05). Likewise, 100 μM of 5-FU and 8 Gy of radiation treatment also significantly inhibited expression of both of these CSC markers. Notably, the results have shown that combination treatment was more effective in inhibiting CSCs as compared to individual treatment of either drug alone or radiation alone.

**Fig. 6:**
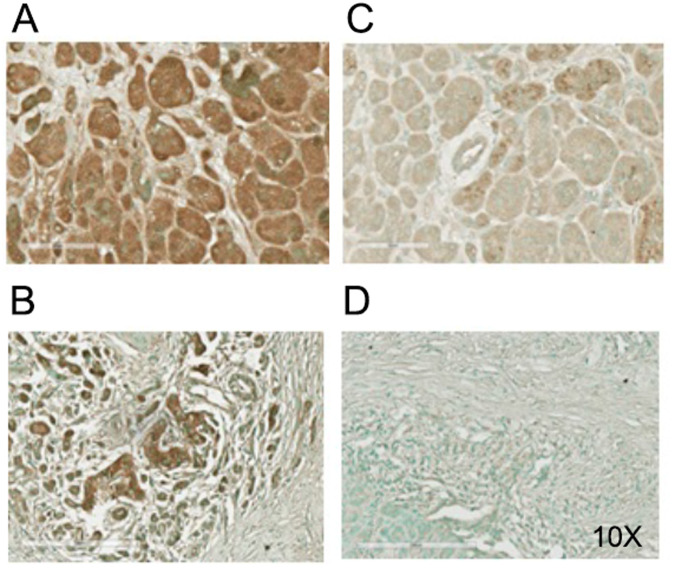
IHC staining of OCT4 and SOX2 in normal and tumor pancreatic tissues of cancer patients. **A** Staining of OCT4 in normal pancreatic tissues. **B** OCT4 staining in pancreatic tumor tissues. **C** SOX2 expression in normal pancreatic tissues. **D** SOX2 expression in pancreatic tumor tissues.

Based on our findings, we conclude that combination therapy may be more effective in eradicating CSCs to treat pancreatic cancer as compared to single therapy. Further, we also monitored OCT4 and SOX2 markers expression in normal pancreatic, and patient’s derived tumor tissues using immunohistochemistry (IHC) staining. The heterogeneous and poorly differentiated expression of OCT4 (Fig. 6A, B) and SOX2 (Fig. 6C, D) was found in tumor tissues, as compared to homogenous and well differentiated expression in normal pancreas. These results suggest that heterogenous expression of OCT4 and SOX2 could be linked to poor prognosis in pancreatic cancer patients and resistant to therapies.

### Activation and involvement of β-catenin signaling in chemo-radiation resistance in pancreatic cancer

**Fig. 7:**
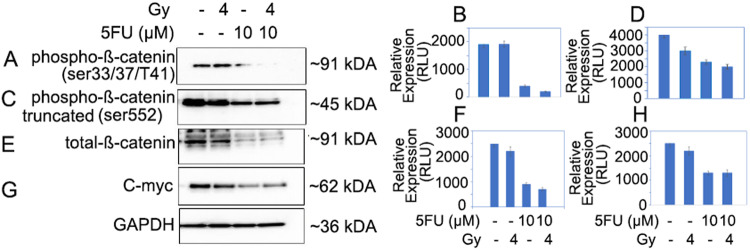
Inhibition of β-catenin pathway in tumor organoids treated with 5-FU, radiation and combination of both (*p = <0.05). **A**, **B** Inhibition of phosphorylated (S33/37/T41) β-catenin and its quantification in tumor organoids; lane 1, untreated control; lane 2, organoids treated with 4 Gy radiation; lane 3, organoids treated with 10 µM 5-FU; lane 4, organoids treated with combination of 10 µM 5-FU and 4 Gy radiation. **C**, **D** Inhibition of phosphorylated (S552) β-catenin in nucleus and its quantification in tumor organoids; lane 1, untreated control; lane 2, organoids treated with 4 Gy radiation; lane 3, organoids treated with 10 µM 5-FU; lane 4, organoids treated with combination of 10 µM 5-FU and 4 Gy radiation. **E**, **F** Inhibition of total β-catenin and its quantification in tumor organoids; lane 1, untreated control; lane 2, organoids treated with 4 Gy radiation; lane 3, organoids treated with 10 µM 5-FU; lane 4, organoids treated with combination of 10 µM 5-FU and 4 Gy radiation. **G**, **H** Inhibition of cMyc expression in nuclear extracts and quantification in tumor organoids.

In order to study the effect of chemotherapy and radiation therapy, both alone and in combination, on the β-catenin signaling, tumor organoids were treated with either 10 μM of 5-FU, 4 Gy of RT, or a combination of both (10 μM of 5-FU for 12 h followed by 4 Gy of RT). After treatment, the organoids were incubated at 37 °C for 4 h to observe changes in β-catenin and its downregulatory signals. The results showed that β-catenin was significantly inhibited in the nuclear fraction of tumor organoids treated with 10 μM 5-FU, and in combination with 4 Gy of RT, compared to the untreated control. There was also a significant inhibition of phosphorylated β-catenin (S33/S37/T41) in tumor organoids treated with the combination of 5-FU and radiation (8%) (Fig. 7A, B; lane 4). Furthermore, β-Catenin (S552) was significantly inhibited (<50%) in tumor organoids treated with the combination of 10 μM of 5-FU and 4 Gy of radiation compared to the untreated control (Fig. 7C, D; lane 4). These results suggest that phosphorylated β-Catenin (S552) in the nucleus was inhibited by 5-FU, and in combination with RT, it was unable to perform its transcriptional function. Similarly, we observed inhibition of total β-Catenin (Fig. 7E, F; lanes 3 and 4), as well as its downstream cMyc (Fig. 7G, H; lanes 3 and 4), in the nuclear fraction of tumor organoids treated with 5-FU and RT. Furthermore, we also identified GSK3B in tumor organoids treated with 4 and 8 Gy of radiation (Supplementary Fig. 1). The exact molecular mechanism behind chemotherapy and radiation resistance in CSCs is not yet completely understood. However, initial research suggests that β-catenin pathways may play a crucial role in causing radiation and chemoresistance in pancreatic CSCs. Overall, the data strongly suggest that the β-catenin pathway is involved in chemotherapy and radiation resistance. Notably, the combination of 5-FU and radiation resulted in significant inhibition of the β-catenin pathway in cancer stem cells in pancreatic tumor organoids.

## Discussion

Pancreatic tumor organoids derived from primary tumor is an attractive model system to examine treatment response to anticancer drugs and radiation before initiating the treatment in the clinic. In this study, tumor organoids showed a response to drug and radiation treatment similar to that of in vivo tumors, which could help in the treatment of patient tumors [14, 15]. We examined the treatment response of tumor organoids to 3-BP and radiation by Optical Metabolic Imaging (OMI). The treatment response of tumor organoids was recorded as the change in the redox ratio of NADH and FAD by autofluorescence [16]. The results showed that tumor organoids treated with a combination of 100 µM of 3-BP and 4 Gy of radiation experienced 94.7% inhibition in OMI, leading to the death of these organoids compared to the untreated control (Fig. 1A, B). Thus, tumor organoids could serve as an attractive model to evaluate tumor response to chemo-radiation treatment [14, 17].

Locally advanced PC is a deadly disease with high levels of intra-tumoral hypoxia, which causes chemotherapy and radiation resistance by protecting CSCs. Pancreatic CSCs are present in hypoxic niche in the tumor which are capable of self-renewal and play an important role in therapeutic resistance and treatment failure [7, 8]. In the present study, we have observed significant upregulation of OCT4 and SOX2 expression in tumor organoids treated with radiation indicating the stemness features associated with therapeutic resistance [18, 19]. Studies have shown that the overexpression of OCT4, and SOX2 in pancreatic tumor organoids treated with 4 and 8 Gy of radiation can suppress the generation of reactive oxygen species (ROS), promote DNA repair in tumor cells [20] and can lead to resistance to radiation therapy. Previous studies have also found that upregulation of OCT4 can cause resistance to radiotherapy by modulating the EMT process, which is strongly associated with tumor invasion and migration, leading to poor prognosis in patients with rectal cancer [10, 21, 22]. The aberrant upregulation of SOX2 has also been implicated in CSCs self-renewal and resistance to therapies in osteocarcoma and lung cancer patients [23] and could be an attractive target for anti-cancer therapy [24]. Additionally, in a recent report, SOX2 and OCT4 have been implicated in poor prognosis of renal cell carcinoma [25], and their coexpression contributed to immunosuppressive phenotype [26]. Our results also showed that 5-FU a chemotherapy drug down regulated the expression of OCT4 and SOX2 in tumor organoids which was further confirmed by Flow Cytometry data. Thus, previous reports also corroborate our finding that 5-FU inhibits cancer stemness by inhibiting phosphorylation of P38 and MAPK proteins which play an important role in stemness maintenance of tumors leading to cell cycle arrest and tumor cell death [27–30].

**Fig. 8:**
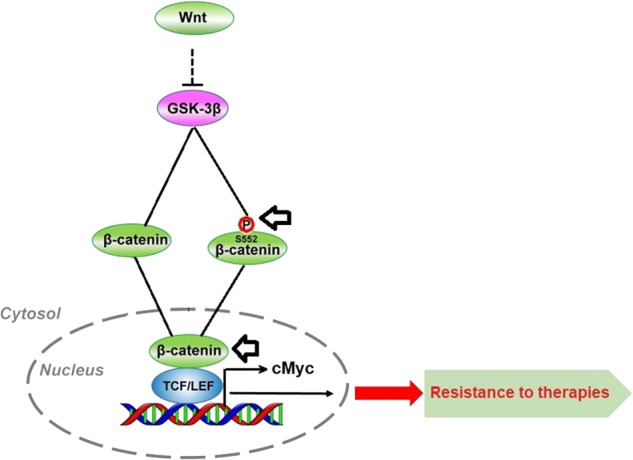
Inhibition of β-catenin pathway in CSC of pancreatic tumor organoids.

Furthermore, based on the experimental evidence, it has been demonstrated that a combination of 5-FU and radiation can effectively inhibit the immunofluorescence of OCT4 and SOX2 CSC markers in tumor organoids, compared to a single treatment modality. The combination treatment, therefore, can potentially target multiple active molecular pathways in cancer cells, making it an effective approach to treating locally advanced PC [31, 32]. Noticeably, the data has shown inhibition of the β-catenin pathway in chemo-radiation treatment in pancreatic tumor organoids, and the combination of 5-FU and RT effectively inhibited both total and nuclear β-Catenin, underscoring impact of combination treatment (Fig. 8).

In conclusion, the data unambiguously suggest that OCT4 and SOX2 cancer stem cells markers are upregulated by radiation treatment and could be playing an important role in radiation resistance. The combination of 5-FU chemotherapy drug and radiation could be an effective treatment option to kill cancer stem cells in PC which might be responsible for therapy resistance and recurrence of PC. Furthermore, pancreatic tumor organoids could serve as a model to investigate the role of CSCs which could be an attractive target for developing pancreatic cancer therapy.

## Material and methods

### Culture of tumor organoids and its characterization

Pancreatic tumor organoids were grown from tumor tissues after euthanizing animals following UMB, School of Medicine IACUC protocol (1019006). Tumors were excised and washed with fresh DMEM/F12 medium and processed for organoid production as described earlier [14]. The finely minced tumor tissues were digested in Tissue Digestion Cocktail (Collagenase IV (3.75 ml), Dispase (3.75 ml) and DNase I (1 mg/ml) in DMEM/F12 (22.2 ml) with 15 mM HEPES) for 20 min. The digested tissue fragments were allowed to settle by gravity for 5 min and supernatant was carefully removed and passed through 70 µm strainer (Stem Cell Tech). The pass through was discarded, and tissues and ductal fragments were collected in 10 ml chilled DMEM/F12 in 15 ml sterile falcon tube. Subsequently, tubes were centrifuged at 300 X g for 5 min. Each pellet was suspended into 25 µl chilled Matrigel (Corning, USA), mixed 5–8 times with cold pipette tip, and cultured as dome in a 24 well plate prewarmed overnight. The plates with Matrigel domes were then incubated at 37 °C for 10 min with 5% CO2. After the domes solidified, 0.5 mL pancreatic cult media containing 2.5 ng/ml recombinant EGF, 2.5 ng/ml FGF, (R&D Systems), 2% v/v vitamin B27, 200 μg/ml ALK5 inhibitor, 0.021 mg/ml gastrin, 81.5 mg/ml N-acetylcysteine, 122 mg/ml Nicotinamide, 1:1000 Y-27632 (Rho-kinase inhibitor) (Sigma) in 100 ml DMEM/F12 GlutaMAX (ThermoFisher Scientific, Waltham, MA) was added to each well. For coculturing, cancer-associated fibroblasts (CAFs) were isolated from tumor tissues and cultured in 60 mm cell culture dish in DMEM/F12 media with 5% FBS. After three passages, CAFs were mixed with organoids in a 1:4 ratio and cultured in Matrigel dome in DMEM/F12 minimal media lacking noggin, TGF-β inhibitor and vitamin B27. The organoid culture was maintained in the incubator for 3-4 days to allow organoids to grow. On day four, the plates were treated, and organoid growth was monitored using an EVOS cell imaging system (ThermoFisher Scientific; Waltham, MA) at 4x magnification under bright-field light condition for 10 days following treatment.

### Treatment of organoids with anti-tumor drugs and RT

For radiation treatment delivery, the co-cultured organoids were irradiated at 2.3 Gy/min using an Xrad 320 biological irradiator (Precision X-Ray; Madison, CT) operating at 320 kVp, 2-mm Al filtration, and 13 mA (1-mm Cu half-value layer). Samples were placed at 50-cm source-to-surface distance on a foam surface to eliminate backscatter. Organoids were irradiated in an AP direction, creating geometric conditions closely matching those used for reference dosimetry. Irradiators for both in vivo tumor and organoids irradiations were calibrated using a NIST-traceable PTW TN30013 Farmer-type ionization chamber and a PTW Unidose electrometer (PTW Freiburg; Breisgau, Germany) following the American Association of Physicists in Medicine (AAPM) Task Group 61 in-air calibration protocol for X-ray irradiators [33].

To evaluate the proliferation and response of organoids to fractionated RT, organoids were treated with doses of 4, and 8 Gy. Irradiation treatments were delivered in fractions of 2 Gy/fraction, with one fraction delivered per day on subsequent days until the entire dose was delivered. Moreover, to study effect of drug 3BP treatment, organoids were treated with 100 µM concentration (IC_50_) for 24 h and growth response was monitored and recorded by bright field imaging. In addition, to study the combinatorial dose response of 3BP with RT, organoids were first treated with 100 µM of 3BP for 24 h, followed by 4 Gy RT (delivered in two fractions as described above). After all treatments, the media was changed, and organoids were allowed to grow for 10 days. For each treatment, eight (*n* = 8) organoids were studied. RT and drug induced changes in organoids morphology was recorded once a day (every 24 h) for 10 days after chemo treatment or delivery of the final fraction of RT treatment using brightfield imaging obtained at ×4 and ×10 magnification with an EVOS microscope (ThermoFisher Scientific, Waltham, MA).

### Immunofluorescence and metabolic imaging

Organoids were grown in 24 well plates in 50% Matrigel (untreated or treated as described above) were fixed in 10% formalin for 12 h, washed with 1× PBS for three times, and treated with 0.2% triton X-100 in 1× PBS for 5 min. The triton x-100 was removed and incubated with blocking reagent (5% goat serum, 1% bovine serum albumin) in 1× PBS for 1 h. The blocking reagent was removed, and organoids were incubated with either: 1) a primary antibody anti-mouse OCT4 (BioLegend, USA) in a 1:100 dilution, or 2), SOX2 also in 1:100 dilution (Cell Signaling Technology; Danvers, MA) in a cold room overnight. The next day, antibody was removed and washed with 1X twin-buffered saline with tween (TBST) for 5 min each for three times. Then organoids were incubated with fluorescently labeled secondary antibody Alexa fluor 488 (ThermoFisher Scientific; Waltham, MA) in a 1:400 dilution. After washing with 1X TBST, organoids were incubated with Hoechst dye 1 μg/ml for 15 min and washed with 1× PBS three times. Finally, 1× PBS was added to the labeled organoids, and fluorescence images were acquired with green and 4′,6-diamidino-2-phenylindole (DAPI) acquisition fields. Organoids derived from pancreatic tumors were grown under 3D environments and subjected to different doses of radiation therapy (RT) and chemo-RT. Optical Metabolic Imaging (OMI) was performed at various time points after treatment, including 0, 24, 48, 72, 96, 120 and 144 h. The NADH/FAD ratio of treated and untreated tumor organoids were measured by sequentially acquiring NADH and FAD images for the same field of view to record autofluorescence due to changes in tumor cell metabolism. The OMI was acquired using a Zeiss 7MP upright multiphoton confocal microscope equipped with a 20×1.0 NA dipping objective. A femtosecond pulsed laser was used to excite at 750 nm, and emission was collected at 460–500 nm with GaAsP PMTs. The treatment with radiation (RT) and 3-Bromopyruvate prevented the oxidation of NADH to NAD+ in the electron transport chain. Consequently, an excess of NADH led to an increase in the optical redox ratio and NADH and FAD fluorescence. Thus, optical metabolic endpoints distinguish the treated tumor organoids from the untreated. ImageJ software was used to analyze images and quantify treatment response to drugs and radiation [34].

### Immunoblotting

After drug treatment for 48 h tumor organoids were harvested and transferred in 1.5 ml tube, and incubated on ice for 15 min. The organoids were pelleted by centrifugation at 1000 rpm for 5 min, and then washed with cold 1 X PBS and suspended in 1X RIPA buffer. Organoids were sonicated for 30 s, pulse 01, amplitude 50% using a Sonicator (Fisher scientific, USA). The sonicated organoids were kept on ice for 15 mins, transferred to 1.5 ml tubes and centrifuged at 12000 rpm for 15 min at 4 °C. The supernatant was collected, and protein concentration was measured using Pierce BCA protein assay kit (ThermoFisher Scientific, USA). Immunoblot analysis was performed as described previously [35].

### Flow cytometry analysis of cancer stem cell markers

Tumors organoids treated with chemo and radiation treatment were digested with digestion cocktail and converted to single cell suspensions for flow cytometry analysis [35]. Single cells were stained with OCT4 (Cat # NB100-2379), and SOX2 (Cat # 4900 s) antibodies with recommended concentrations for 30 min at 4 °C in 100 μl FACS buffer. All antibodies were from BioLegend (San Diego, CA, USA). All flow cytometry analysis was performed at the University of Maryland Greenebaum Comprehensive Cancer Center Flow Cytometry Shared Services. Flow cytometry acquisition was performed using an LSRII instrument (BD Biosciences, USA) and data were analyzed using FlowJo software (Version 10.6, Tree Star Inc., Ashland, OR, USA).

### Immunohistochemistry staining of OCT4 and SOX2 in human pancreatic tissues

Human pancreatic tumor tissue id # 19779, 18600, and normal pancreas tissue id # 19988, 18421were acquired from Pathology Core at University of Maryland Medical Center under IRB # HP-00096381. We preserved tumor tissues in 10% formalin and sandwiched in cassette for paraffin embedding. The embedding, tissue sectioning and slide preparation were carried out in histopathology core facility, University of Maryland, Baltimore. Tissue section were cut in 5 μM thickness. Unmasking and antibody staining were performed according to the antibody provider Cell Signaling Technology (USA), and Novus biologicals, (USA) and we used antigen unmasking citrate-based PH 6.0, Vectastain universal quick kit Vector Laboratories, (USA). After blocking with 2.5% horse serum provided in kit, (vector stain) for 1 h at room temperature, the slides were incubated with OCT4 antibodies in 1:200 dilution, (Novus biologicals, USA) and SOX2 antibodies in 1:200 (Cell Signaling Technology, USA) dilution in 1.25% blocking buffer. Briefly, after unmasking and tissue endogenous peroxidase inactivation we incubated the slides in 3% hydrogen peroxide for 10 min, the slides were then blocked as described above. Subsequently, the slides were incubated with the primary antibodies at 4 °C in cold room for overnight. Next, the slides were washed with 1X TBST for 5 min with two more repetitions. Secondary antibody incubation, and brown color development, were performed using ABC Vectastain universal quick kit, and DAB substrate (Vector Laboratories, USA). Counter stain was performed with Methyl green from Vector Lab (USA). The slides were clear with xylene, dehydrate with ethanol and mounted with cytoseal 60 (ThermoFisher Scientific USA). Images were captured with Evos microscope color at 10 X (ThermoFisher Scientific USA).

### Statistical analysis

Tumor organoids response to drugs and radiation treatment was determined from the difference in autofluorescence (changes in OMI) of control organoids, and the organoids subjected to a single or combination treatment. Since each comparison involved only two data groups [(1) untreated control and (2)] individual or combination treatment type], the statistical significance of the difference between untreated control organoids, and individual or combined treatment type organoids was calculated using an unpaired one-sided student t-test (*P* value) [36]. Differences in autofluorescence were considered significant for *P* < 0.05 or lower.

### Supplementary information

Supplementary Figure 1

## Data Availability

The research data files will be available upon request.
