# Supplementary figures and images for: SOX2 and OCT4 mediate radiation and drug resistance in pancreatic tumor organoids
Source: Cell Death Discov. 2024 Mar 1;10:106. doi: 10.1038/s41420-024-01871-1 (PMC10907757; doi:10.1038/s41420-024-01871-1)

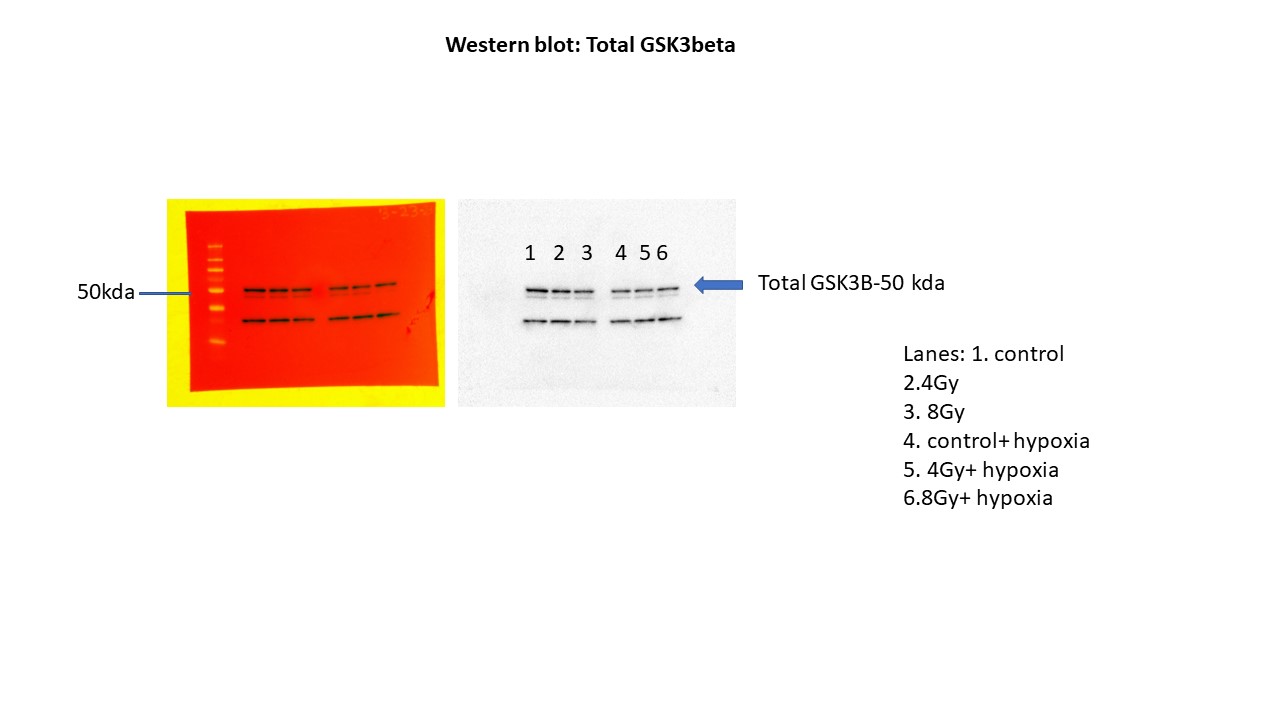

Supplement: Supplementary file 1 — Supplementary Figure 1 [file 41420_2024_1871_MOESM1_ESM.jpg]
